# Genome Sequence of a *Providencia stuartii* Strain Isolated from *Lucilia* *sericata* Salivary Glands

**DOI:** 10.1128/genomeA.00250-17

**Published:** 2017-04-27

**Authors:** Ye Yuan, Yu Zhang, Shuhua Fu, Tawni L. Crippen, David K. Visi, M. Eric Benbow, Michael S. Allen, Jeffery K. Tomberlin, Sing-Hoi Sze, Aaron M. Tarone

**Affiliations:** a Department of Computer Science and Engineering, Texas A&M University, College Station, Texas, USA; b Department of Biochemistry and Biophysics, Texas A&M University, College Station, Texas, USA; c Southern Plains Agricultural Research Center, Agricultural Research Service, USDA, College Station, Texas, USA; d Department of Molecular and Medical Genetics, University of North Texas Health Science Center, Fort Worth, Texas, USA; e Department of Entomology and Department of Osteopathic Medical Specialties, Michigan State University, East Lansing, Michigan, USA; f Department of Entomology, Texas A&M University, College Station, Texas, USA

## Abstract

We present here the draft genome sequence of a *Providencia stuartii* strain, derived from the salivary glands of larval *Lucilia sericata*, a common blow fly important to forensic, medical, and veterinary science. The genome sequence will help dissect coinfections involving *P. stuartii* and *Proteus mirabilis*, as well as blow fly–bacteria interactions.

## GENOME ANNOUNCEMENT

*Providencia stuartii* is a Gram-negative bacillus bacterium (1) that frequently causes urinary tract infections in hospital patients and has intrinsic resistance to antibiotics (2). Infections have been reported to progress to bacteremia (3), diarrhea (4), peritonitis (5), meningitis (6), infective endocarditis (7), and often co-occur with *Proteus* infections (8). Additionally, *Providencia* species were reported to associate with blow flies (9, 10), stable flies (11), Mexican fruit flies (12), vinegar flies (13), as well as house flies (14). *Providencia* can cause variable infections and different levels of mortality in their hosts (13) and have been shown to impact blow fly attraction to resources (15).

The *P. stuartii* strain Crippen was isolated from *Lucilia sericata*, a green bottle fly, which is of importance to decomposition ecology (18) as related to the medical and forensic sciences (16, 17). Our strain was coisolated along with *Proteus mirabilis* strain WOOD (19), which can affect *L. sericata* attraction to, and colonization of, resources (20, 21). Mixed microbial communities, including *Proteus-Providencia* coinfections, which increase the incidence of bacteremia and urolithiasis (8), can have properties distinct from those of their individual components (22, 23). This property of mixed cultures has also been shown to impact fly behavior and life history (24). Therefore, knowledge of this genome will help elucidate fly–microbe interactions that are important to forensic science and ecology, as well as coinfections relevant to medicine.

Here, we present a draft genome of *P. stuartii* strain Crippen. The genomic sequence was isolated from a colony derived from maggot salivary glands of *L. sericata* third instars raised on beef liver. DNA sequencing was performed using an Ion Torrent personal genome machine (Life Technologies, Inc., Carlsbad, CA, USA) after preparation with a NEBNext fast DNA fragmentation library prep set. The sequencing data comprise 1,383,989 reads, with an average length of 212 bp, totaling 294 Mb. Sequence assembly using the PATRIC full assembly service (25) produced 243 contigs, with an *N*_50_ of 191,375 bp and a total of 4.72 million nucleotides, resulting in approximately 62-fold coverage. This strain is comparably similar to three previously sequenced *P. stuartii* strains: MRSN 2154 (GenBank accession no. CP003488.1), ATCC 33672 (GenBank accession no. CP008920.1), and FDAARGOS_145 (GenBank accession no. CP014024.1). Further genome assemblies based on CONTIGuator (26) indicate that the longest 37 contigs were mapped to the reference strain, with 93.8% of the assembled nucleotides aligned to the *P. stuartii* MRSN 2154 genome. No evidence of plasmids was found based on analysis with the PlasmidFinder version 1.3 server (27).

Whole-genome annotation was generated using the NCBI Prokaryotic Genome Annotation Pipeline (28) using the PATRIC assembly draft genome. The annotated genome contains 4,534 genes, including 4,428 protein-coding regions, eight 5S rRNAs, 14 16S rRNAs, 15 23S rRNAs, 60 tRNAs, and nine ncRNAs. There were also 600 pseudogenes annotated. A total of eight prophage regions were identified with PHAST (29), of which five regions predicted to be intact, two regions incomplete, and one region that is of questionable functionality. Strain-specific gene functions and phage insertions will be useful in elucidating the interactions among *L. sericata*, *P. mirabilis*, and *P. stuartii*.

### Accession number(s).

This whole-genome shotgun project has been deposited at DDBJ/ENA/GenBank under the accession number LVIE00000000. The version described in this paper is the first version, LVIE01000000.
